# Endothelial ADAM10 controls cellular response to oxLDL and its deficiency exacerbates atherosclerosis with intraplaque hemorrhage and neovascularization in mice

**DOI:** 10.3389/fcvm.2023.974918

**Published:** 2023-01-27

**Authors:** Emiel P. C. van der Vorst, Sanne L. Maas, Kosta Theodorou, Linsey J. F. Peters, Han Jin, Timo Rademakers, Marion J. Gijbels, Mat Rousch, Yvonne Jansen, Christian Weber, Michael Lehrke, Corinna Lebherz, Daniela Yildiz, Andreas Ludwig, Jacob F. Bentzon, Erik A. L. Biessen, Marjo M. P. C. Donners

**Affiliations:** ^1^Department of Pathology, Cardiovascular Research Institute Maastricht (CARIM), Maastricht University Medical Center, Maastricht, Netherlands; ^2^Institute for Molecular Cardiovascular Research (IMCAR), RWTH Aachen University Hospital, Aachen, Germany; ^3^Interdisciplinary Centre for Clinical Research (IZKF), RWTH Aachen University Hospital, Aachen, Germany; ^4^Institute for Cardiovascular Prevention (IPEK), Ludwig Maximilian University of Munich, Munich, Germany; ^5^German Centre for Cardiovascular Research (DZHK), Partner Site Munich Heart Alliance, Munich, Germany; ^6^Department of Molecular Genetics, Cardiovascular Research Institute Maastricht (CARIM), Maastricht University Medical Center, Maastricht, Netherlands; ^7^Department of Medical Biochemistry, Amsterdam UMC, Locatie AMC, Amsterdam, Netherlands; ^8^Department of Biochemistry, Cardiovascular Research Institute Maastricht (CARIM), Maastricht University Medical Center, Maastricht, Netherlands; ^9^Department of Internal Medicine I, RWTH Aachen University Hospital, Aachen, Germany; ^10^Institute of Molecular Pharmacology, RWTH Aachen University Hospital, Aachen, Germany; ^11^Institute of Experimental and Clinical Pharmacology and Toxicology, PZMS, ZHMB, Saarland University, Homburg, Germany; ^12^Experimental Pathology of Atherosclerosis Laboratory, Spanish National Center for Cardiovascular Research (CNIC), Madrid, Spain; ^13^Atherosclerosis Research Unit, Department of Clinical Medicine, Aarhus University, Aarhus, Denmark

**Keywords:** a disintegrin and metalloproteinase 10, atherosclerosis, endothelial cells, intraplaque hemorrhage, neovascularization, inflammatory signaling, LOX-1

## Abstract

**Introduction:**

The transmembrane protease A Disintegrin And Metalloproteinase 10 (ADAM10) displays a “pattern regulatory function,” by cleaving a range of membrane-bound proteins. In endothelium, it regulates barrier function, leukocyte recruitment and angiogenesis. Previously, we showed that ADAM10 is expressed in human atherosclerotic plaques and associated with neovascularization. In this study, we aimed to determine the causal relevance of endothelial ADAM10 in murine atherosclerosis development *in vivo*.

**Methods and results:**

Endothelial *Adam10* deficiency (*Adam10*^ecko^*)* in Western-type diet (WTD) fed mice rendered atherogenic by adeno-associated virus-mediated PCSK9 overexpression showed markedly increased atherosclerotic lesion formation. Additionally, *Adam10* deficiency was associated with an increased necrotic core and concomitant reduction in plaque macrophage content. Strikingly, while intraplaque hemorrhage and neovascularization are rarely observed in aortic roots of atherosclerotic mice after 12 weeks of WTD feeding, a majority of plaques in both brachiocephalic artery and aortic root of *Adam10^ecko^* mice contained these features, suggestive of major plaque destabilization. *In vitro*, *ADAM10* knockdown in human coronary artery endothelial cells (HCAECs) blunted the shedding of lectin-like oxidized LDL (oxLDL) receptor-1 (LOX-1) and increased endothelial inflammatory responses to oxLDL as witnessed by upregulated ICAM-1, VCAM-1, CCL5, and CXCL1 expression (which was diminished when *LOX-1* was silenced) as well as activation of pro-inflammatory signaling pathways. LOX-1 shedding appeared also reduced *in vivo*, as soluble LOX-1 levels in plasma of *Adam10^ecko^* mice was significantly reduced compared to wildtypes.

**Discussion:**

Collectively, these results demonstrate that endothelial ADAM10 is atheroprotective, most likely by limiting oxLDL-induced inflammation besides its known role in pathological neovascularization. Our findings create novel opportunities to develop therapeutics targeting atherosclerotic plaque progression and stability, but at the same time warrant caution when considering to use ADAM10 inhibitors for therapy in other diseases.

## 1. Introduction

Atherosclerosis is a lipid-driven chronic inflammatory disease, which manifests in regions with disturbed flow in the medium- and large-sized arteries. The initiation of atherosclerosis is characterized by endothelial cell activation and dysfunction, leading to the disruption of the endothelial barrier function and the active recruitment of leukocytes into the vessel wall (1).

A crucial mechanism to regulate cell signaling and subsequent cellular responses, like cell recruitment, is proteolytic processing of transmembrane proteins, also referred to as (ectodomain) shedding. The A Disintegrin And Metalloproteinase (ADAM) family is involved in the shedding of numerous cell surface proteins, e.g., adhesion molecules, chemokines and cytokine receptors (2). One of the most prominent ADAM family members, ADAM10, has previously been reported to be implicated in several physiological and pathological processes, e.g., tumor growth and metastasis (2, 3). It is ubiquitously expressed, constitutively active in all vascular cells and deficiency of *Adam10* results in embryonic lethality at stage E9.5, when the vascular system develops (4). Indeed, and as a master regulator of Notch, ADAM10 is well known to have an important role in cardiovascular development (5, 6) and to limit sprouting angiogenesis and pathological neovascularization in developing mouse retina (7–9). Additionally, ADAM10 can regulate endothelial permeability and leukocyte transmigration by cleaving vascular endothelial cadherin (VE-Cadherin) (10, 11) and intracellular adhesion molecule-1 (ICAM-1) (12). ADAM10 has a large repertoire of substrates, many of which are involved in the pathogenesis of atherosclerosis, including lectin-like oxidized low-density lipoprotein (oxLDL) receptor 1 (LOX-1) (3, 13). Previously, we have shown that ADAM10 is expressed in human atherosclerotic arteries, correlating with plaque progression (11). In a conditional knockout mouse model, we demonstrated that deficiency of *Adam10* in myeloid cells did not affect atherosclerotic lesion size, but enhanced plaque stability by increasing fibrosis (14). *ADAM10* was also expressed in plaque endothelium and associated with neovascularization (11). However, whether endothelial ADAM10 contributes in the pathogenesis of atherosclerosis or this increased expression reflects a disease-related response, e.g., an attempt to dampen/limit plaque neovascularization has not been investigated so far.

Here, we used the model of adeno-associated virus-mediated proprotein convertase subtilisin/kexin type 9 (PCSK9) gene transfer in mice deficient in endothelial *Adam10* (15–17), to investigate the causal role of endothelial ADAM10 in atherosclerosis development. We report that the absence of endothelial ADAM10 severely aggravates atherosclerotic plaque formation in mice. Moreover, while plaque granulocyte and collagen content remained unchanged, lesions of mice with endothelial *Adam10* deficiency had larger necrotic cores and concomitant lower macrophage content. Strikingly, lesions from *Adam10* deficient mice contained intraplaque microvessels and showed clear signs of intraplaque hemorrhage. These features have never been observed before at such early time point (12 weeks) in murine aortic root atherosclerotic lesions of Western type diet (WTD) fed mice (18). Knockdown of *ADAM10* in human coronary artery endothelial cells (HCAECs) challenged with oxidized low-density lipoprotein (oxLDL) resulted in a more severe pro-inflammatory, hence more pro-atherogenic, phenotype, in agreement with significantly reduced LOX-1 shedding.

## 2. Materials and methods

### 2.1. Animals

Mouse experiments were approved by the Animal Ethics Committee of Maastricht University, Netherlands (permit number 2013-009), and were performed in compliance with the Dutch government guidelines. Female endothelial specific (*Tie2-Cre*) *Adam10* knockout (*Adam10^ecko^*) and wildtype (*Adam10^wt^*) littermate control mice on a mixed genetic background were previously described (7) and generously provided by Dr. C. Blobel (New York, USA).

### 2.2. Baseline vessel morphometry

Left carotid arteries were collected from female wildtype and *Adam10^ecko^* mice (aged 10–12 weeks; *n* = 7 and 8, respectively), fixed overnight in 1% paraformaldehyde and embedded in paraffin. Cross-sections (4 μm) were cut and four sections (100 μm apart) were stained with Movat’s stain to visualize the elastic laminae. Pictures were taken using a Leica DM3000 light microscope and sections were analyzed in a blinded manner using computerized morphometry (Leica QWin V3). For each mouse, total vessel area (area within external elastic lamina), medial area (area between internal and external elastic lamina), and lumen area (area within internal elastic lamina) were measured at 100 μm distance from the aortic arch.

### 2.3. Atherosclerotic lesion induction and analysis

Female wildtype and *Adam10^ecko^* mice (aged 10–12 weeks; *n* = 16 and 14, respectively) were rendered prone to atherosclerosis by a single intravenous injection of adeno-associated virus serotype 8 containing D377Y-murine PCSK9 [AAV-PCSK9; 1 × 10^11^ vector genomes per mouse; as described previously (15)], followed by western type diet (WTD) feeding (0.25% cholesterol; Special Diets Services, Witham, Essex, UK). Blood was collected from the tail vein for analyses of plasma lipids at baseline (before WTD) and after 1, 3, 6, 9, and 12 weeks of WTD feeding after 4 h fasting. After 12 weeks of WTD feeding, mice were anesthetized, euthanized, and perfused with PBS containing nitroprusside (0.1 mg/ml, Sigma-Aldrich, Seelze, Germany). Mouse hearts and the brachiocephalic trunk (BC) of the right carotid artery were excised and fixed overnight in 1% paraformaldehyde. Serial paraffin sections of the aortic root were cut (4 μm) and stained with hematoxylin and eosin (H&E, Sigma) for morphometric analysis of lesion size, plaque, necrotic core area (defined as acellular regions) and plaque phenotype staging. Total plaque areas were obtained by averaging morphometric measurements of five representative H&E sections (20 μm apart) of the aortic root and five representative H&E sections (20 μm apart) of the BC. Plaque phenotype characterization was determined as previously described (19), with slight modifications. Plaques were classified as early (foam cell rich, but lacking a necrotic core), moderately advanced (containing a fibrotic cap and often a necrotic core, but no medial macrophage infiltration) and advanced lesions, typified by medial macrophage infiltrates, elastic lamina degradation and more pronounced necrosis and fibrosis. For each mouse, the aortic valves (three per mouse) was scored by an experienced pathologist in a blinded manner based on above described characteristics using a 0–5 scale (0–1: early, 2–3: moderate, 4–5: advanced; each valve was scored on the cross sections with the most advanced plaque stage) and these results were used to determine the percentages of the different stages. Martius, Scarlet and Blue (MSB) staining was used for semi-quantitative analyses of fibrin deposits (severity score of 1–5, average of 5 sections). Atherosclerotic lesions were further characterized for macrophage (MAC3, clone M3/84, 1:200, BD Biosciences, New Jersey, USA), granulocyte (Ly6G, clone 1A8, BD) and collagen (Sirius Red, Sigma) content. Minimal cap thickness was measured at the thinnest point of the fibrous cap. A polarization filter and birefringence color discrimination were used to differentiate various collagen structures (ranging from loosely patched—immature–thin collagen, to tightly packed—mature–thick collagen fibers), as described by MacKenna et al. (20). Additionally, sections were analyzed for microvessels (CD31, clone MEC13.3, 1:25, BD), apoptosis [Cleaved Caspase-3 (Asp175, 1:100, Cell Signaling #9661)], and alpha smooth muscle actin (αSMA; clone 1A4, 1:3000, Sigma). Cell nuclei were counterstained with hematoxylin. Immunofluorescent staining of erythrocytes in atherosclerotic lesions was performed using anti-TER-119 antibodies (1:500, Biolegend); cell nuclei were stained with DAPI. Pictures were taken using a Leica DM3000 light microscope and sections were analyzed in a blinded manner using computerized morphometry (Leica QWin V3). Necrotic core was defined as cell and nucleus free plaque area, containing cholesterol clefts.

### 2.4. Blood lipid analyses

Blood was collected at the start (*t* = 0) and after 1, 3, 6, 9, and 12 weeks of WTD. Plasma was separated by centrifugation (2100 × *g*, 10 min, 4°C), and stored at -80°C until further use. Plasma cholesterol and triglycerides were determined using standard enzymatic kits (Cholesterol FS’10; Triglycerides FS 5′ Ecoline; Diagnostic Systems GmbH, Holzheim, Germany) according to the manufacturer’s instructions.

### 2.5. Cell culture

Human Primary Coronary Artery Endothelial Cells (HCAECs; CC-2585), EGM-2 Bulletkit medium (CC-3162) and ReagentPack Subculture Reagents (CC-5034) were purchased from Lonza. Cells were kept at 37°C and 5% CO_2_ under sterile conditions in a humidified incubator. Subculturing and medium refreshing were performed according to the manufacturer’s protocol. For *ADAM10* silencing studies, cells were transfected with either 20 nM negative control siRNA (Negative Control DsiRNA, Cat. nr. 51-01-14-03, Integrated DNA Technologies) or 20 nM ADAM10 siRNA duplex mix (TriFECTa^®^ Kit DsiRNA Duplex, Integrated DNA Technologies) consisting of the following duplexes: hs.Ri.ADAM10.13.1 (5′-rArUr CrArCrUrUrCrArArGrArArGrUrArArArGrCTA-3′ and 5′rUrArGr CrUrUrUrArCrUrUrCrUrArGrUrUrGrArArGrUrGrArUrGrU-3′), hs.Ri.ADAM10.13.2 (5′-rGrUrCrArUrGrUrUrArArArGrCrGrArUr UrGrArUrArCrAAT-3′ and 5′-rArUrUrGrUrArUrCrArArUrCrGr CrUrUrUrArArCrArUrGrArCrUrG-3′), and hs.Ri.ADAM10.13.3 (5′-rCrArUrGrGrUrGrArArArCrGrCrArUrArArGArArUrCrAAT-3′ and 5′-rArUrUrGrArUrUrCrUrUrArUrGrCrGrUrUrUrCrArCrC rArUrGrArA-3′). For *LOX-1* silencing studies, cells were transfected with either 20 nM negative control siRNA (Negative Control DsiRNA, Cat. nr. 51-01-14-03, Integrated DNA Technologies) or 20 nM LOX-1 siRNA (Silencer Select, Ambion). Transfection was performed *via* siPORT™ NeoFX™ Transfection Agent according to the manufacturer’s protocol (Invitrogen by Life Technologies). Cells were transfected for 24 h (for double knockdown of *ADAM10* and *LOX-1*, both siRNA complexes were combined) and the transfection efficiency was evaluated by qPCR. Cells were optionally treated with 25 μg/ml oxLDL 24 h before evaluating the impact of *ADAM10* silencing.

### 2.6. Flow cytometry analyses

Absolute circulating leukocyte subset numbers were determined by flow cytometry calibrated using Trucount Beads (BD). Blood was collected at the start (*t* = 0) and after 12 weeks of WTD. Erythrocytes were removed by incubation with erylysis buffer (155 mM NH_4_Cl and 10 mM KHCO_3_). Leukocytes were defined as CD45^+^ (Biolegend), T-lymphocytes as CD45^+^ CD3^+^ (eBioscience) NK1.1^–^ (BD), NK cells as CD45^+^ CD3^–^ NK1.1^+^, B-lymphocytes as CD45^+^ CD3^–^ NK1.1^–^ B220^+^ (BD) granulocytes as CD45^+^ CD3^–^ NK1.1^–^ B220^–^ CD11b^+^ (BD) Ly6G^+^ (BD), and monocytes as CD45^+^ CD3^–^ NK1.1^–^ B220^–^ CD11b^+^ Ly6G^–^. Data were acquired using a FACS Canto II (BD Bioscience) and analyzed with FACSdiva software (BD Bioscience).

To measure protein expression of vascular adhesion molecule-1 (VCAM-1) and ICAM-1 on the cell surface of HCAECs, flow cytometric analysis was performed 24 h after transfection with ADAM10 siRNA (with or without LOX-1 siRNA). Cells were also simultaneously stimulated with or without 25 μg/ml oxLDL. The HCAECs were then harvested and stained for VCAM-1 (eBioscience) and ICAM-1 (eBioscience).

### 2.7. RNA isolation and cDNA synthesis

Total RNA isolation from cell culture samples was performed by commercially available RNA isolation kit from Zymoresearch (Direct-zol microprep kit) according to the manufacturer’s protocol. The quality (A_260_/A_280_) and the quantity (ng/μL) of the RNA was measured by NanoPhotometer N60/N50 (Implen). A ratio of ∼2 for A_260_/A_280_ was accepted as good quality RNA. RNA samples were diluted to the same concentration and the cDNA synthesis was performed *via* the commercially available iScript cDNA synthesis kit from Bio-Rad according to the manufacturer’s protocol.

### 2.8. PCR

Quantitative real-time PCR was performed using PowerUp™ SYBR™ Green Master Mix (Life Technologies), according to the manufacturer’s protocol. Real-time PCR reactions with the primer pair for *ADAM10* (5′TTGCCTCCTCCTAAACCACTTCCA-3′ and 5′AGGCAGTAGGAAGAACCAAGGCAA-3′) or for *ADAM17* (5′ GGGAAGTGACTTAGCAGATG-3′ and 5′CTAGATTCACCTTCA CCTTACC-3′) were performed using the ViiA7 Real Time PCR system (Life Technologies). Gene expression was normalized to Beta Actin according to the ΔΔCt method.

### 2.9. RNA preparation and sequencing

Snap frozen aortic arches from atherosclerotic *Adam10^wt^* and *Adam10^ecko^* mice were pooled (three pools of two aortic arches) and RNA was extracted as described above. Library construction was done by using Oligo(dT) magnetic beads to select mRNA with poly(A) tail or hybridize the rRNA with DNA probe and digest the DNA/RNA hybrid strand, followed by DNase I reaction to remove DNA probe. After purification, the target RNA was obtained. The target RNA and reverse transcription was fragmented to double-strand cDNA (dscDNA) by N6 random primer, which was followed by end repair of the dscDNA with phosphate at 5′ end and stickiness “A” at 3′ end, and ligation and adaption with stickiness “T” at 3′ end to the dscDNA. Two specific primers are used to amplify the ligation product, and the PCR product was denatured by heat and the single-strand DNA is cyclized by splint oligo and DNA ligase followed by sequencing on the prepared library. Samples were sequenced by BGISEQ-500 system.

### 2.10. RNA-sequencing data analysis

Raw sequences were aligned to the murine reference cDNA (GRCm39.v107) obtained from the Ensembl using kallisto (v0.48.0) (21). Transcript abundances were aggregated into gene level by the R package tximport (v1.22.0) (22). Genes with an average read count below 5 were removed. In addition, only protein-coding genes were included, resulting in 14,980 genes for differential expression analysis.

Based on the raw read counts, gene differential expression analysis was performed by the R package DESeq2 (v1.34.0) (23) with the parameter alpha set as 0.05. The Benjamini–Hochberg procedure was used to adjust the *p*-values to decrease the false discovery rate. We set log2 fold change > 0 (up-regulation) or < 0 (down-regulation) as well as a significance level of adjusted *p*-value = 0.05 as the thresholds for significantly differentially expressed genes (DEGs). Raw read counts were normalized using the variance stabilizing transformation provided in the DESeq2 package for heatmap visualization.

We associated gene differential expression with biological functions by gene set overrepresentation analysis (GSOA) and gene set enrichment analysis (GSEA) (24). For GSOA, we analyzed the significantly up-/down-regulated genes separately to associated DEGs with Gene Ontology annotation terms. GSEA was performed on the list of all analyzed genes sorted based on log2 fold change from high to low based on hallmark gene sets obtained from the Molecular Signatures Database. Both analyses were performed using the R package clusterprofiler (v4.2.2) (25). *P*-values were corrected by the Benjamini–Hochberg procedure.

The raw sequencing data analyzed in this study have been deposited into the Gene Expression Omnibus (GEO) with the accession number GSE209602.

### 2.11. ELISA

LOX-1, CCL5, and CXCL1 levels in supernatant were measured with the LOX-1/OLR1 Human ELISA kit (ThermoFisher), ELISA Deluxe set human CCL5 (Biolegend) and Human GROa uncoated ELISA kit (ThermoFisher), respectively. LOX-1 was measured in murine plasma using the Mouse LOX-1/OLR1 ELISA kit (ThermoFisher).

### 2.12. Kinase activity profiling

Kinase profiles were determined using the PamChip^®^ peptide based tyrosine kinase (PTK) and the PamChip^®^ Ser/Thr Kinase (STK) microarray system on PamStation^®^12 (PamGene International, ‘s-Hertogenbosch, Netherlands). Each PTK-PamChip^®^ array contains 196 individual phospho-site(s) that are peptide sequences derived from substrates for Tyrosine kinases. Each STK-PamChip^®^ array contains 144 individual phospho-site(s) that are peptide sequences derived from substrates for Ser/Thr kinases. Peptide phosphorylation is visualized by detection of the fluorescent signal emitted after binding of the FITC-conjugated antibody to the phosphorylation site. HCAECs were transfected and stimulated with/without oxLDL as described above and subsequently washed once in ice-cold PBS, with 4 biological replicates per condition, and lysed for 15 min on ice using M-PER Mammalian Extraction Buffer containing Halt Phosphatase Inhibitor and EDTA-free Halt Protease Inhibitor Cocktail (1:100 each; Thermo Fischer Scientific). Lysates were centrifuged for 15 min. at 16.000 × *g* at 4°C in a pre-cooled centrifuge. Protein quantification was performed with Pierce™ Coomassie Plus (Bradford) Assay according to the manufacturer’s instructions.

For the PTK assay, 10.0 μg and for the STK assay, 2.0 μg of protein was applied per array (*N* = 4 per condition) and carried out using the standard protocol supplied by Pamgene International B.V. Images were recorded by a CCD camera PamStation^®^12. The spot intensity at each time point was quantified (and corrected for local background) using the BioNavigator software version 6.3 (PamGene International, ’s-Hertogenbosch, Netherlands). Upstream Kinase Analysis (UKA) (26), a functional scoring method (PamGene) was used to rank kinases based on combined specificity scores (based on peptides linked to a kinase, derived from 6 databases) and sensitivity scores (based on treatment-control differences).

Over-representation analyses (ORA) of the Kyoto Encyclopedia of Genes and Genomes (KEGG) database for the kinases with significant differences from control were performed using the ClusterProfiler R-package (27). The list of kinases of interest contains the kinases with higher Median Final Scores (> 1.2). The *p*-values were adjusted for multiple comparisons by false discovery rate (FDR).

### 2.13. Statistics

Data are expressed as mean ± standard error of the mean (SEM). Statistical analysis was performed using GraphPad Prism version 9.1.1 (GraphPad Software, Inc., San Diego, CA, USA). Outliers were identified using the ROUT = 1 method. Gaussian distribution was tested *via* the D’Agostino-Pearson omnibus normality test, while homogeneity of variance by Levene’s test. Significance was tested using either Student’s *t*-test (with Welch correction as required) or Mann–Whitney *U*-test for normally and non-normally distributed data, respectively, unless stated otherwise. A two-tailed *p*-value < 0.05 was considered statistically significant.

## 3. Results

### 3.1. Endothelial *Adam10* deficiency significantly augments atherosclerosis development

**FIGURE 1:**
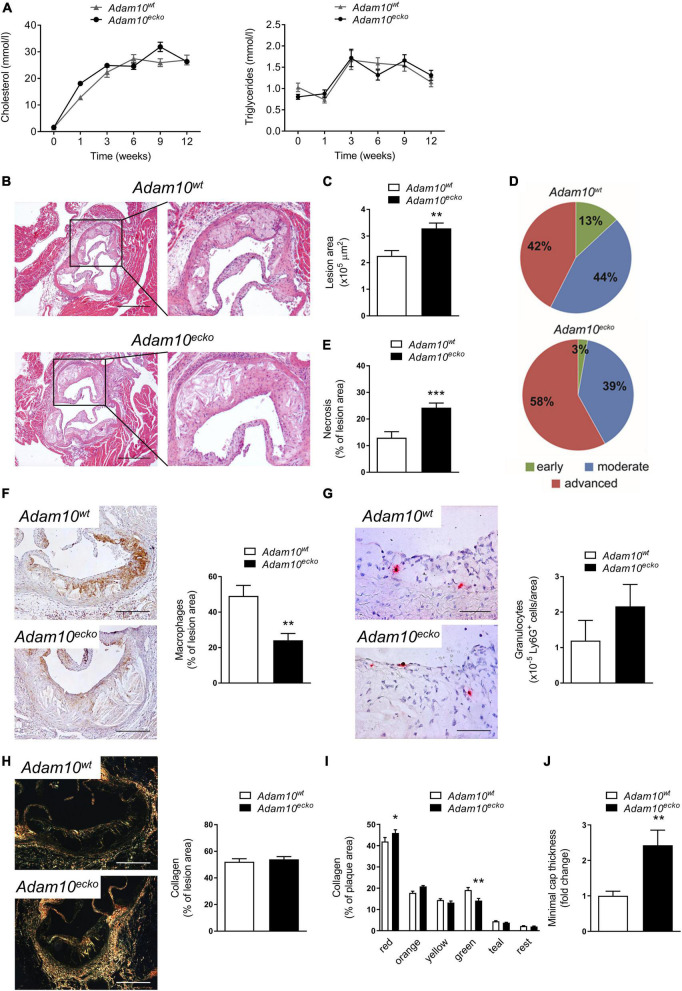
*Adam10^ecko^* significantly increases atherosclerotic lesion formation and intraplaque necrosis. Endothelial *Adam10* deficient (*Adam10^ecko^*) and wildtype mice (*Adam10^wt^*) mice were rendered hyperlipidemic by AAV8-PCSK9 gene transfer, followed by 12 week western type diet feeding. **(A)** Plasma cholesterol and triglyceride levels (*n* = 13–16). **(B–D)** Atherosclerotic lesion area **(B,C)** and plaque stage quantification **(D)** in the aortic root (H&E staining, *n* = 13–15; scale bar, 400 μm). Indicated percentages reflect the total number of valves (three per mouse) that are classified as belonging to the respective plaque stage. **(E)** Quantification of necrotic core area (*n* = 13–16). **(F–H)** Representative images of (immuno)histological stainings for macrophages (**F**, MAC3^+^; *n* = 12–14; scale bar, 200 μm), granulocytes (**G**, Ly6G^+^, *n* = 13–16; scale bar, 100 μm) and total collagen (**H**, Sirius Red imaged with polarized light; *n* = 13–16; scale bar, 200 μm) in aortic root atherosclerotic plaques with quantification. **(I)** Quantification of different collagen fibers in the plaques, ranging from thick–mature collagen (red), to loosely packed–thin collagen fibers (green) (*n* = 13–16). Sidak’s multiple comparison test was conducted to determine the statistical significance between the two groups. **(J)** Quantification of minimal cap thickness (*n* = 13–15). *p ≤ 0.05; **p ≤ 0.01; ***p ≤ 0.001.

To investigate the causal impact of endothelial ADAM10 in atherosclerosis, *Adam10^wt^* and *Adam10^ecko^* mice were rendered atherogenic *via* adeno-associated virus (AAV) aided murine PCSK9 gene transfer (15), followed by WTD feeding for 12 weeks. As expected, WTD feeding resulted in a prominent increase in plasma cholesterol and triglycerides levels, though no differences were observed between both ADAM10 genotypes (Figure 1A). Additionally, endothelial *Adam10* deficiency had no effect on general leukocyte numbers in the blood before or after 12 weeks of WTD feeding (Supplementary Figure 1A). At baseline, i.e., under normolipidemic conditions, there was no indication for any large vessel abnormalities, based on left carotid artery morphometry (Supplementary Figures 1B, C). Furthermore, body weight after 12 weeks of WTD feeding was not changed between groups (Supplementary Figure 1D).

Although ADAM10 expression in human atherosclerosis positively correlates with disease progression and endothelial *Adam10* knockdown is known to reduce leukocyte recruitment (13), endothelial *Adam10* deficiency resulted in a remarkable 45% increase in atherosclerotic plaque size in the aortic root, compared to wildtype controls (Figures 1B, C). Besides this significant increase in plaque size, *Adam10^ecko^* mice also showed a noteworthy increase in plaque progression (Figure 1D). In addition, plaque expansion and progression in *Adam10^ecko^* mice was associated with a significantly increased (50%) relative necrotic core content (Figure 1E), with a concomitant decrease in relative macrophage content (Figure 1F), while the absolute macrophage area and cleaved Caspase-3 area were unchanged (Supplementary Figures 1E, F). In contrast, granulocyte (Figure 1G) and total collagen (Figure 1H) contents were unchanged between both genotypes. Collagen fibers in *Adam10^ecko^* plaques were more mature (Figure 1I) and minimal cap thickness increased (Figure 1J).

**FIGURE 2:**
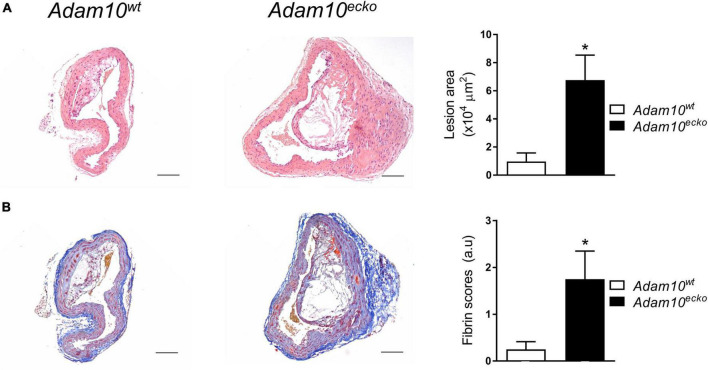
Atherosclerotic lesion size and intraplaque fibrin deposits are increased in *Adam10^ecko^* mice. **(A)** Examples of HE-stained plaques in brachiocephalic trunk and quantification of atherosclerotic lesion area (*n* = 5–7). **(B)** Examples of MSB-stained plaques in brachiocephalic trunk and pathological scoring (*n* = 8) of intraplaque fibrin deposits as evidence for IPH. *p ≤ 0.05.

Interestingly, endothelial *Adam10* deficiency also significantly increased the plaque area in the brachiocephalic trunk of the right carotid artery (BC), a site where lesion development generally is less progressed than in the aortic root (Figure 2A). This is also the only site in mice where advanced lesions incidentally were seen to display signs of plaque rupture or intraplaque hemorrhage (IPH) (18). Interestingly, overt fibrin deposits, reflective of IPH associated thrombus formation, were found in 5 out of 8 *Adam10^ecko^* mice, while only minor fibrin deposits were seen in 2 out of 8 *Adam10^wt^* mice (Figure 2B).

### 3.2. Aortic root plaques of *Adam10^ecko^* mice show overt intraplaque hemorrhage and neovascularization

**FIGURE 3:**
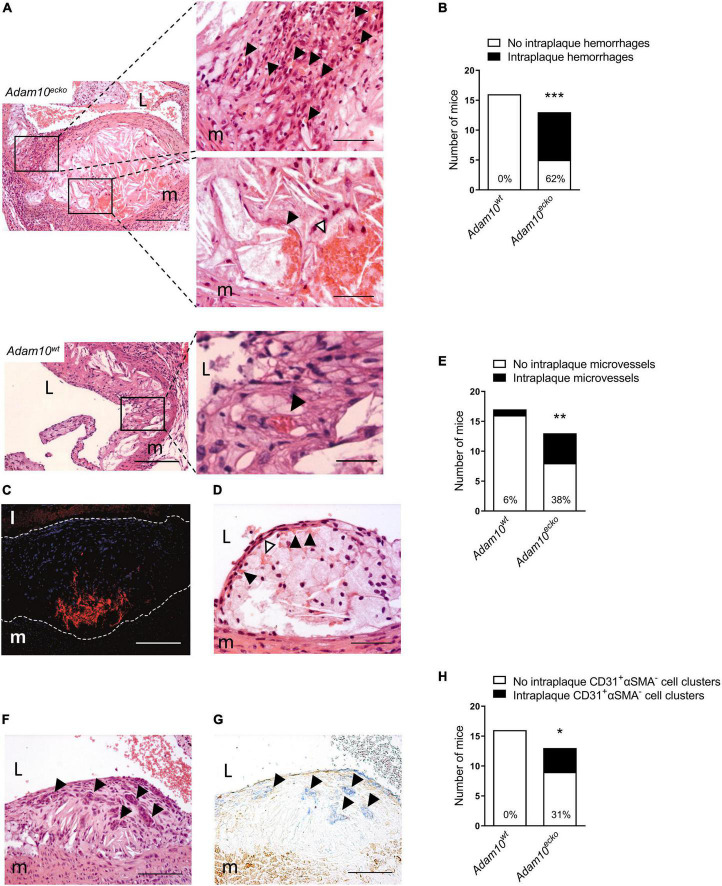
Atherosclerotic lesions of *Adam10^ecko^* mice display several plaque destabilizing features. **(A)** *Adam10^ecko^* left panel: Atherosclerotic lesion in the aortic root containing pronounced intraplaque hemorrhage and microvessels of an *Adam10^ecko^* mouse (H&E, scale bar, 400 μm). *Adam10^ecko^* upper right detail panel: Detail of multiple small microvessels containing erythrocytes (indicated with black arrowheads) in aortic root lesion (H&E, scale bar, 200 μm). *Adam10^ecko^* lower right detail panel: Detail of a large microvessel containing erythrocytes (indicated with black arrowhead) and intraplaque hemorrhage (indicated with white arrowhead) at the plaque base in the aortic root (H&E, scale bar, 50 μm). *Adam10^wt^* left panel: Atherosclerotic lesion in the aortic root of an *Adam10^wt^* mouse containing a microvessel (H&E, scale bar, 400 μm). *Adam10^wt^* right detail panel: Detail of the microvessel containing erythrocytes (indicated with black arrowhead) in aortic root lesion (H&E, scale bar, 50 μm). **(B)** Quantification of intraplaque hemorrhage in aortic root of *Adam10^wt^* and *Adam10^ecko^* mice after 12 weeks on western type diet. **(C)** Immunofluorescence staining of erythrocytes (TER-119, red) in the atherosclerotic intima (cell nuclei, DAPI, blue) of an adjacent slide shown in panel (**A**; scale bar, 100 μm). Lesion borders are indicated with dashed lines. **(D)** Atherosclerotic lesions containing intraplaque hemorrhage (indicated with white arrowheads) possibly originating from neovessels from the luminal side of the aorta (indicated with black arrowheads; H&E, scale bar, 100 μm). **(E)** Quantification of intraplaque microvessels in aortic root of *Adam10^wt^* and *Adam10^ecko^* mice after 12 weeks on western type diet. **(F,G)** Clusters of cells (indicated with black arrowheads) within an atherosclerotic lesion of an *Adam10^ecko^* mouse (**F**, H&E, scale bar, 100 μm), which are positive for CD31 (**G**, blue, indicated by black arrowheads) and negative for α-SMA (G, brown; scale bar, 100 μm). **(H)** Quantification of intraplaque CD31^+^αSMA^–^ cell clusters in aortic root of *Adam10^wt^* and *Adam10^ecko^* mice after 12 weeks on western type diet. L, lumen; m, media. Fisher’s exact test was conducted to determine the statistical significance between the two groups. *P ≤ 0.05; **P ≤ 0.01; ***P ≤ 0.001.

Remarkably, further examination revealed that atherosclerotic plaques in the aortic root from *Adam10^ecko^* mice were also rich in IPH and neovessels, which to our knowledge is the first report at this site, especially at this relatively early time point (12 weeks WTD, i.e., ∼22 weeks of age, Figures 3A, B). This IPH consisted mainly of erythrocytes (in contrast to the fibrin deposits observed in the brachiocephalic artery) was confirmed by TER-119 staining for erythrocytes (Figure 3C) and shown to be present in a striking 62% of *Adam10^ecko^* mice, significantly higher compared to *Adam10^wt^* mice, which did not contain any hemorrhages at all (Figure 3B). IPHs were not only observed near the abluminal side of the intima (Figures 3A, C), but also more closely to the lumen (Figure 3D). Intraplaque microvessels, which are most likely functional based on the presence of intraluminal erythrocytes, were sixfold more frequent in aortic root plaques of *Adam10^ecko^* mice (Figures 3A, E). Moreover, aortic root plaques of *Adam10^ecko^* mice contained intraplaque CD31^+^αSMA^–^ cell clusters, which may represent (immature) vessels or sites of endothelial-to-mesenchymal transition (28) (Figures 3F–H). While ADAM10 is known to control angiogenic processes in a non-atherosclerotic setting (7–9), these observations clearly indicate not only a protective role for ADAM10 in plaque neovascularization, but also in atherosclerosis development and progression, suggesting a crucial role of endothelial ADAM10 in maintaining endothelial quiescence/homeostasis.

### 3.3. Atherosclerotic plaques from *Adam10^ecko^* mice demonstrate a more inflamed phenotype

**FIGURE 4:**
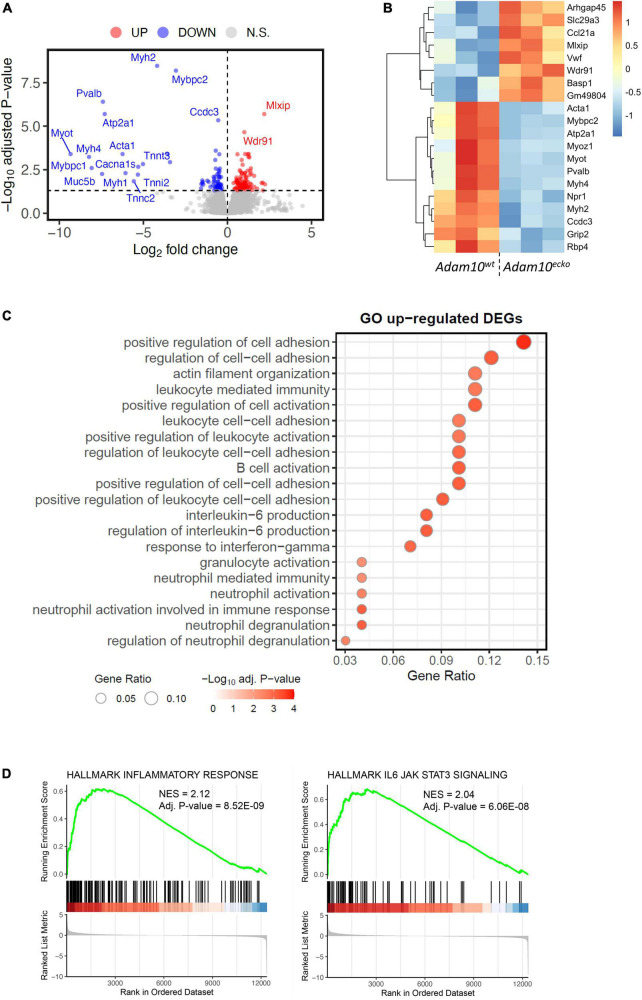
RNA-sequencing demonstrates an inflammatory genetic profile in vessels from *Adam10^ecko^* mice. **(A)** Volcano plot visualizing up-/downregulated genes in *Adam10^ecko^* vs. *Adam10^wt^* mice. Horizontal line represents adjusted *p*-value of 0.05, while vertical lines represent log2FC of 0. N.S., not-significant. **(B)** Heatmap of top 20 most differentially expressed genes. Variance stabilizing transformed expression values are z-normalized per gene/row. **(C)** Gene ontology of vessels from *Adam10^ecko^* vs. *Adam10^wt^* mice evaluated by GSOA. **(D)** GSEA figures showing the dysregulation of inflammatory responses and IL6-JAK-STAT3 signaling in *Adam10^ecko^* vs. *Adam10^wt^* mice.

In order to examine the underlying mechanisms by which endothelial specific *Adam10* deficiency affects the atherosclerotic vessel, we mapped the changes in transcriptional makeup by bulk RNA-sequencing of aortic arches from atherosclerotic *Adam10^ecko^* and *Adam10^wt^* mice. We detected 181 differentially expressed genes (DEGs), of which 103 genes were significantly up- and 78 genes downregulated upon endothelial *Adam10* deficiency (Figures 4A, B). Albeit bulk-sequencing has limitations over single-cell plaque analysis (29) as it cannot specify the cell-origin of the detected genes, we did detect well-known endothelial specific genes, e.g., von Willebrand factor, which is a known marker of endothelial dysfunction involved in platelet and leukocyte adhesion (30, 31), to be highly upregulated in plaques of *Adam10^ecko^* mice. Interestingly, although not specific for endothelium, another highly upregulated gene Arhgap45 has been suggested to negatively regulate endothelial barrier function (32). Over-representation analysis (ORA) revealed enrichment of GO pathways involved in inflammation, immune cell activation and cell adhesion in *Adam10^ecko^* mice (Figure 4C), of which the latter process involves DEGs both relevant for leukocyte adhesion to endothelium as well as endothelial integrity and cell-cell adhesion. In line with the GSOA results, GSEA also clearly demonstrates an increase in inflammatory response and inflammatory signaling pathways like IL-6-JAK-STAT3 signaling in plaques from *Adam10^ecko^* mice compared to *Adam10^wt^* mice (Figure 4D). Combined, these results suggest that mice with an endothelial *Adam10* deficiency have plaques with a more inflamed phenotype.

### 3.4. *ADAM10* silencing induces pro-atherogenic phenotype in HCAECs upon oxLDL stimulation

**FIGURE 5:**
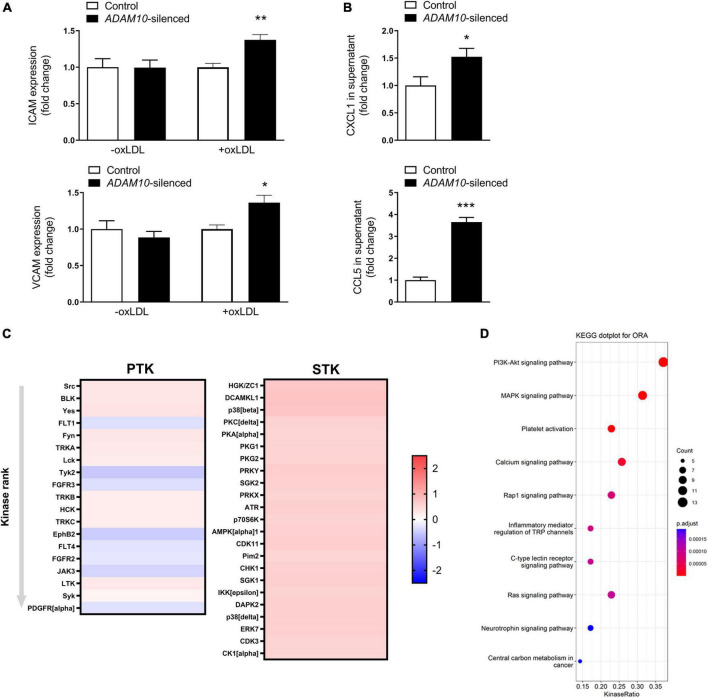
*ADAM10* silencing results in a pro-inflammatory, pro-atherogenic phenotype in HCAECs. **(A)** Surface expression of ICAM and VCAM, measured by flow cytometry in control and *Adam10* silenced HCAECs stimulated with or without 25 μg/ml oxLDL for 24 h (*n* = 3–6). Fold change has been determined for each condition by comparing to the respective control condition. **(B)** Quantification of soluble CCL5 and CXCL1 in supernatant of control and *ADAM10* silenced HCAECs stimulated with 25 μg/ml oxLDL for 24 h, measured by ELISA (*n* = 8–11). **(C)** Kinomic analysis of control and *ADAM10* silenced HCAECs stimulated with 25 μg/ml oxLDL for 30 min. Visualized is the heatmap of kinases that are up-regulated upon *ADAM10* silencing after oxLDL stimulation. Red color reflects increased phosphorylation, while blue color reflects decreased phosphorylation in *ADAM10* silenced HCAECs (fold change compared to scrambled control cells, *n* = 4). **(D)** Dot plot for over-representation analysis (ORA) of KEGG with significant differences from control using the PTK and STK dataset. Visualized are the top 10 of upregulated pathways in *ADAM10* silenced HCAECs, stimulated with 25 μg/ml oxLDL for 30 min. *p ≤ 0.05; **p ≤ 0.01; ***p ≤ 0.001.

To further investigate the underlying mechanisms by which ADAM10 affects endothelial cells in an atherogenic environment, we silenced *ADAM10* in HCAECs (Supplementary Figure 2A), which did not result in a compensatory upregulation of its closely related family member *ADAM17* (Supplementary Figure 2B), and challenged them with pro-atherogenic oxLDL. Since the adhesion molecules ICAM-1 and VCAM play an important role in atherogenesis and are known substrates of ADAM-proteases (12, 33), the effect of *ADAM10* silencing on their surface expression was evaluated. Though ADAM10 was previously shown to regulate ICAM-1 cleavage (12), at least in TNF-stimulated human umbilical vein endothelial cells, we did not find differences in unstimulated cells. However, oxLDL exposure led to increased ICAM-1/VCAM-1 surface expression (Figure 5A), suggesting an enhanced response to oxLDL in *ADAM10* deficient endothelial cells. The more pro-inflammatory and pro-atherogenic phenotype of oxLDL-stimulated HCAECs after silencing *ADAM10* was further substantiated by the observed increased secretion of the chemokines CXCL1 and CCL5 compared to control oxLDL treated HCAECs (Figure 5B). Furthermore, kinomic analysis confirmed that *ADAM10* silencing induced pro-inflammatory signaling in oxLDL treated HCAECs, exemplified by amongst others increased phosphorylation of p38 and Src (Figure 5C). Furthermore KEGG analysis demonstrated that *ADAM10* silencing results in increased PI3K-Akt and MAPK signaling, known downstream pathways of LOX-1 and mediators of ICAM and chemokine expression in mouse aortic endothelial cells (34, 35) (Figure 5D).

### 3.5. The inflammatory and pro-atherogenic phenotype of endothelial cells lacking *ADAM10* is LOX-1 dependent

**FIGURE 6:**
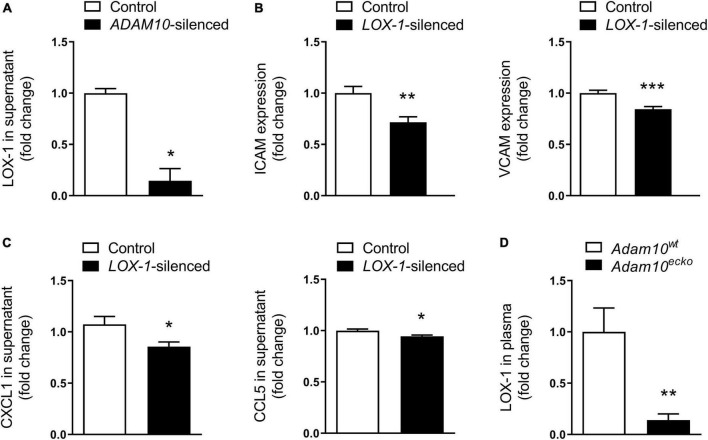
LOX-1 shedding is a key mediator in the observed pro-inflammatory and pro-atherogenic effects by endothelial ADAM10. **(A)** Quantification of soluble LOX-1 in supernatant of control and *ADAM10* silenced HCAECs stimulated with 25 μg/ml oxLDL for 24 h, measured by ELISA (*n* = 3). **(B)** Surface expression of ICAM and VCAM, measured by flow cytometry in control and *LOX-1* silenced HCAECs (all conditions were additionally *ADAM10* silenced) stimulated with or without 25 μg/ml oxLDL for 24 h (*n* = 9). **(C)** Quantification of soluble CCL5 and CXCL1 in supernatant of control and *LOX-1* silenced HCAECs (all conditions were additionally *ADAM10* silenced) stimulated with 25 μg/ml oxLDL for 24 h, measured by ELISA (*n* = 15–22). **(D)** Plasma LOX-1 levels in *Adam10^ecko^* and *Adam10^wt^* mice (*n* = 11–14). *p ≤ 0.05; **p ≤ 0.01; ***p ≤ 0.001.

Since the observed inflammatory effects in HCAECs upon *ADAM10* silencing seem to be dependent on oxLDL stimulation, we evaluated the shedding of the endothelial oxLDL receptor LOX-1, which is a known substrate of ADAM10 (36), previously also shown to be involved in pro-inflammatory responses to oxLDL in mouse aortic endothelial cells (35). Indeed, *ADAM10* silencing strongly decreased LOX-1 shedding in HCAECs, as evidenced by a decrease in soluble LOX-1 in the cells’ culture medium (Figure 6A). To investigate whether LOX-1 is indeed causally involved in the observed inflammatory and pro-adhesion effects, we silenced *LOX-1* in combination with *ADAM10* in HCAECs. In line with our expectation, *LOX-1* silencing significantly reduced ICAM and VCAM expression after oxLDL stimulation of *ADAM10*-silenced HCAECs (Figure 6B). Additionally, also the secretion of CXCL1 and CCL5 in response to oxLDL stimulation is significantly reduced upon *LOX-1* silencing (Figure 6C). Importantly, we could confirm that LOX-1 shedding is also affected by endothelial *Adam10* deficiency *in vivo*, as soluble LOX-1 levels in the plasma were significantly reduced in *Adam10^ecko^* mice (Figure 6D). In summary, reduced LOX-1 shedding in ADAM10-deficient endothelium may render these mice more susceptible to oxLDL-induced inflammatory processes and atherosclerosis.

## 4. Discussion

This study is the first to establish that endothelial *Adam10* deficiency significantly exacerbates atherosclerotic lesion formation and promotes plaque vulnerability in mice, with increased necrosis, IPH and neovascularization as most frequent features. These findings are in line with previous literature showing vascular abnormalities and increased (pathological) neovascularization in endothelial *Adam10* deficient mice (7–9). However, they are in sharp contrast to the significantly reduced atherosclerosis development in mice with endothelial deficiency of *Adam17*, a family member with a large overlap in substrate repertoire (37).

Especially the observation of substantial IPHs and neovascularization in *Adam10^ecko^* mice was to our knowledge unprecedented at this site and age of mice. In previous studies IPH and intraplaque microvessels were only detected in the carotid or brachiocephalic artery of aged (> 40 weeks) atherosclerosis-prone mice (38), upon prolonged WTD feeding of *Apoe*^–/–^ mice (> 40 weeks) (39), after surgical (40), pharmacological [focal mast cell activation (41)] intervention or with haploinsufficiency of a key extracellular matrix component (fibulin-1) (42). IPH has already been identified as critical factor in necrotic core expansion and plaque growth (43), features that we have also observed in *Adam10^ecko^* mice. The decreased relative lesional macrophage content and necrotic core expansion are therefore likely the resultants of the more advanced plaque progression stage in *Adam10^ecko^* mice. Whether this is causal in or secondary to the overt presence of intraplaque microvessels, which are known to provide a major portal for lipids, leukocytes and cholesterol-rich erythrocytes into the atherosclerotic plaque (38, 44, 45), remains to be addressed. Besides this novel observation of tissue neovascularization under chronic inflammatory conditions, several lesions from *Adam10^ecko^* mice also contained CD31^+^αSMA^–^ cell clusters resembling hyperplastic endothelial cells, which were previously only observed in the intestines and kidneys of *Adam10^ecko^* mice (7).

The broad pattern regulatory function of ADAM10 makes it challenging to pinpoint a single responsible key mediator for the observed phenotype in *Adam10^ecko^* mice. Angiogenesis, the sprouting of new blood vessels from the existing vasculature, is a tightly regulated process which is induced by various stimuli, including pro-angiogenic growth factors, like vascular endothelial growth factor (VEGF), hypoxia, metabolic stress and inflammation (46). ADAM10 has already been shown to be able to regulate the angiogenic process through shedding of various key receptors, including VEGF receptor 2 (VEGFR2) and Notch (9, 11, 47). ADAM10-mediated Notch cleavage, for example, is a crucial step in Notch activation, which in endothelial cells limits tip cell selection and sprout formation, thereby reducing excessive sprouting and branching (48). Interestingly, interference with Notch signaling phenocopies the vascular abnormalities caused by ADAM10 deletion under baseline conditions (7–9), suggesting that reduced Notch signaling most likely underlies the pathological neovascularization in atherosclerotic lesions caused by endothelial *Adam10* deficiency.

A second important mechanism potentially contributing to the profound plaque phenotype of endothelial *Adam10* deficiency involves ADAM10’s inflammation dampening functions. As highlighted in this and other studies (12), endothelial ADAM10 plays an important role in the cleavage of adhesion molecules, necessary for efficient transmigration. Thereby, a lack of ADAM10 results in increased and prolonged leukocyte adhesion to the vascular wall, though eventually does not restrict leukocyte transmigration (12). Our RNAseq data indeed confirm that *Adam10^ecko^* atherosclerotic plaques are more inflammatory, with an enrichment in inflammatory signaling, immune cell activation and cell adhesion, which could reflect both leukocyte adhesion and angiogenic processes. Previously, we have demonstrated that endothelial ADAM10 is responsible for the shedding of LOX-1, thereby increasing the surface expression of this pro-atherogenic receptor for oxLDL (34, 35). It has already been shown in fibroblasts that increased *Lox-1* expression results in an increased expression of ICAM-1 and VCAM (49) and also in mouse aortic endothelial cells, enhanced LOX-1 signaling upregulated ICAM-1 and increased pro-inflammatory signaling *via* ERK1/2 and p38 (34, 35), we could confirm in HCAECs that *ADAM10* deficiency indeed reduced LOX-1 shedding and enhanced endothelial proinflammatory responses to oxLDL, including p38 activation and chemokine secretion. Interestingly, the fact that ADAM17 appeared not to be involved in LOX-1 cleavage (35) might explain, at least partly, the different impact on atherogenesis between endothelial *Adam10* and *Adam17* deficiency (37).

Taken together, this study demonstrates that endothelial ADAM10 is a protective factor in atherosclerosis, on the one hand by restraining plaque neovascularization and intraplaque hemorrhage, and on the other hand by limiting inflammation. While the ADAM10-Notch axis is a well-known master regulator of neovascularization, this study demonstrates a crucial role of ADAM10 in pro-inflammatory processes in endothelial cells, at least in part by regulating expression of leukocyte adhesion molecules and by controlling receptor availability for pro-atherogenic stimuli, as demonstrated for LOX-1. Although it remains to be determined to what extent endothelial ADAM10 can influence these vulnerable plaque features in humans, its overt expression in intraplaque microvessels in human plaques suggests an active regulatory function in humans as well (11).

## Data availability statement

The raw data supporting the conclusions of this article will be made available by the authors, without undue reservation.

## Ethics statement

The animal study was reviewed and approved by the Animal Ethics Committee of Maastricht University, Netherlands.

## Author contributions

EV, EB, and MD: conceptualization. EV, KT, SM, and MD: methodology. EV, KT, SM, LP, HJ, TR, MG, MR, and YJ: formal analysis and investigation. EV and KT: writing—original draft preparation. EB and MD: writing—review and editing. EV and MD: funding acquisition. CW, ML, CL, DY, AL, and JB: resources. MD: supervision. All authors contributed to the article and approved the submitted version.
