# Supplementary material for: Endothelial ADAM10 controls cellular response to oxLDL and its deficiency exacerbates atherosclerosis with intraplaque hemorrhage and neovascularization in mice
Source: Front Cardiovasc Med. 2023 Jan 27;10:974918. doi: 10.3389/fcvm.2023.974918 (PMC9911417; doi:10.3389/fcvm.2023.974918)
Supplement: Supplementary file 1 [file Data_Sheet_1.pdf]

Supplemental Figure 1

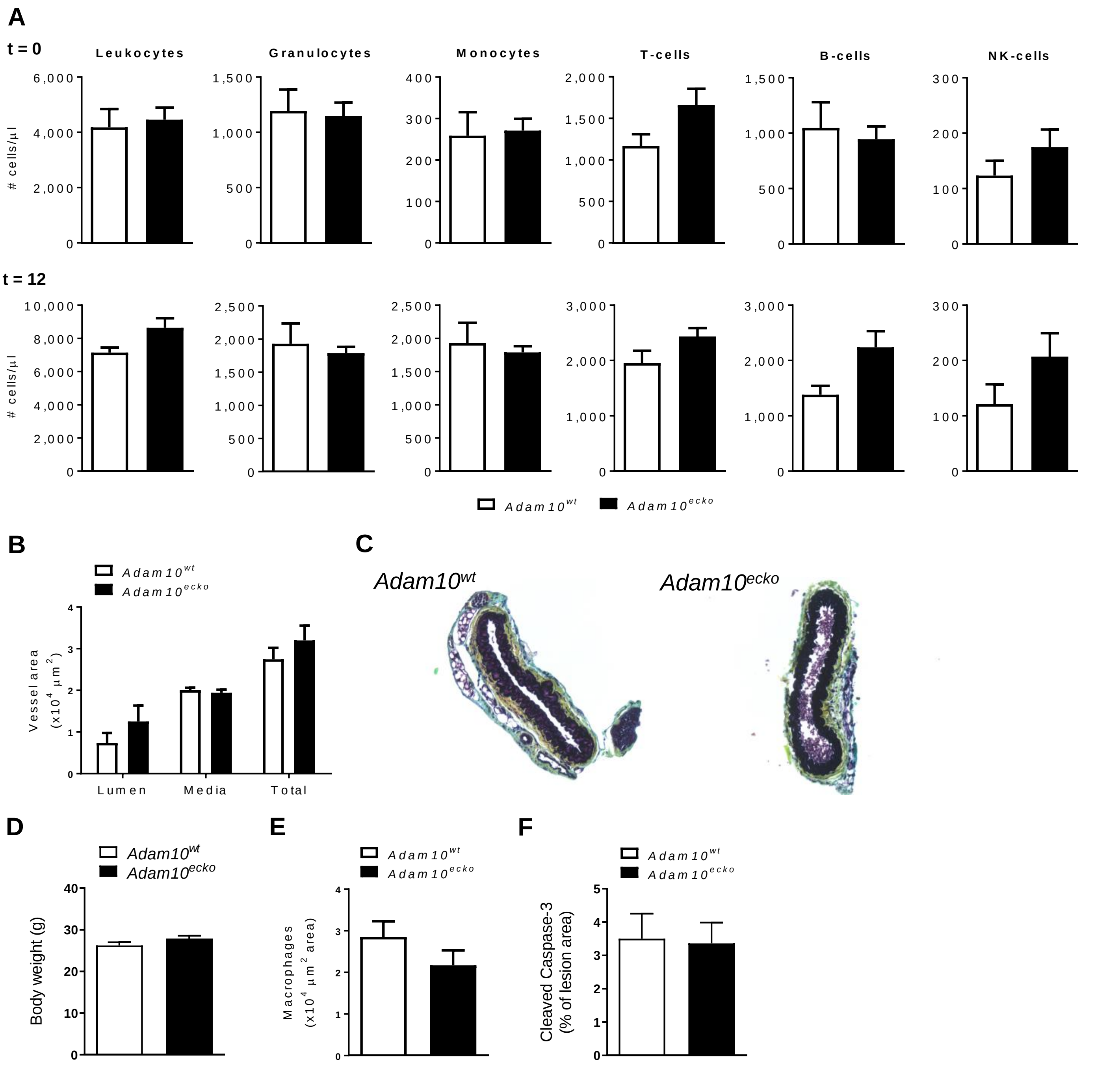

Supplemental figure 1: Endothelial ADAM10 deficiency does not affect blood leukocyte levels. (**A**) Flow cytometry analysis of major leukocyte subsets at baseline and after 12 weeks WTD feeding. (**B-C**) Quantification of large vessel morphology at baseline (B) and representative pictures of left carotid MOVAT staining (C). (**D**) Body weight after 12 weeks WTD feeding. (**E**) Absolute lesional macrophage content in aortic root plaques. (**F**) Cleaved caspase-3 area in aortic root plaques.

Supplemental Figure 2

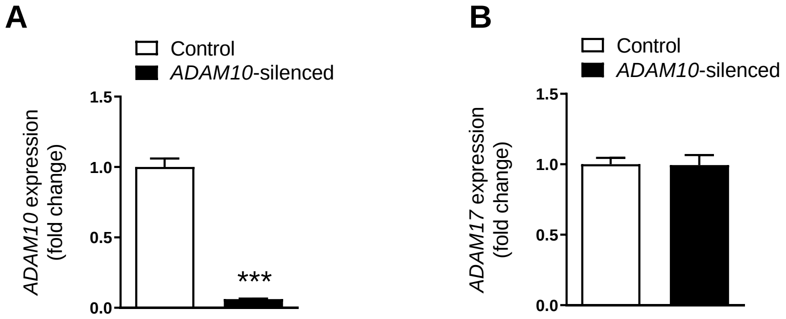

Supplemental figure 2: (**A-B**) Gene expression of *ADAM10* (A) or *ADAM17* (B) in control and *ADAM10* silenced HCAECs (n=6-7).
